# Outcome of Veno-Pulmonary Extracorporeal Life Support in Lung Transplantation Using ProtekDuo Cannula: A Systematic Review and Description of Configurations

**DOI:** 10.3390/jcm13144111

**Published:** 2024-07-14

**Authors:** Massimo Capoccia, Joseph M. Brewer, Mindaugas Rackauskas, Torben K. Becker, Dirk M. Maybauer, Yuriy Stukov, Roberto Lorusso, Marc O. Maybauer

**Affiliations:** 1 South Yorkshire Cardiothoracic Centre, Northern General Hospital, Sheffield Teaching Hospitals NHS Foundation Trust, Sheffield S5 7AU, UK; 2 Nazih Zuhdi Transplant Institute, Specialty Critical Care and Acute Circulatory Support Service, INTEGRIS Baptist Medical Center, Oklahoma City, OK 73112, USA; michael.brewer@integrisok.com; 3 Queen’s University Health Quality Programs, Kingston, ON K7L 3N6, Canada; 4 Department of Surgery, Division of Thoracic Surgery, University of Florida College of Medicine, Gainesville, FL 32610, USA; mindaugas.rackauskas@surgery.ufl.edu (M.R.); yuriy.stukov@medicine.ufl.edu (Y.S.); 5 Department of Emergency Medicine, Division of Critical Care Medicine, University of Florida College of Medicine, Gainesville, FL 32610, USA; t.becker@ufl.edu; 6 Department of Anaesthesiology and Intensive Care Medicine, Philipps University, 35032 Marburg, Germany; dirk.maybauer@staff-uni.marburg.de; 7 Extracorporeal Life Support (ECLS) Centrum, Cardio-Thoracic Surgery and Cardiology Department, Heart & Vascular Center, Maastricht University Medical Center (MUMC), 6229 ER Maastricht, The Netherlands; roberto.lorusso@mumc.nl; 8 Cardiovascular Research Institute (CARIM), 6229 ER Maastricht, The Netherlands; 9 Department of Anesthesiology, Division of Critical Care Medicine, University of Florida College of Medicine, Gainesville, FL 32610, USA; 10 Critical Care Research Group, The Prince Charles Hospital, University of Queensland, Brisbane 4032, Australia

**Keywords:** ECLS, ECMO, extracorporeal membrane oxygenation, lung transplantation, ProtekDuo, right ventricular assist device

## Abstract

**Background:** Refractory end-stage pulmonary failure may benefit from extracorporeal life support (ECLS) as a bridge to lung transplantation. Veno-venous (VV) extracorporeal membrane oxygenation (ECMO) has been recommended for patients who have failed conventional medical therapy and mechanical ventilation. Veno-arterial (VA) ECMO may be used in patients with acute right ventricular (RV) failure, haemodynamic instability, or refractory respiratory failure. Peripheral percutaneous approaches, either dual-site single-lumen cannulation for veno-pulmonary (VP) ECMO or single-site dual-lumen (dl)VP ECMO, using the ProtekDuo right ventricular assist device (RVAD) cannula, has made this configuration a desirable option as a bridge to transplantation. These configurations support the right ventricle, prevent recirculation by placing the tricuspid and pulmonary valve between the drainage and return cannulas, provide the direct introduction of oxygenated blood into the pulmonary artery, and have been shown to decrease the incidence of acute kidney injury (AKI), requiring continuous renal replacement therapy (CRRT) in certain disease states. This promotes haemodynamic stability, potential sedation-weaning trials, extubation, mobilisation, and pre-transplant rehabilitation. **Methods:** A web-based literature search in PubMed and EMBASE was undertaken based on a combination of keywords. The PICOS and PRISMA approaches were used. **Results:** Four case series were identified out of 323 articles, with a total of 34 patients placed on VP ECMO as a bridge to lung transplantation. All relevant data are reviewed and integrated into the Discussion. **Conclusions:** Despite the limited available evidence, the use of ProtekDuo has become very promising for the management of end-stage lung disease as a bridge to lung transplantation.

## 1. Introduction

Lung transplantation has increased in the setting of end-stage lung disease, such as cystic fibrosis, interstitial lung disease, and chronic obstructive lung diseases, often resulting in acute respiratory failure (ARF) and/or acute respiratory distress syndrome (ARDS) [1,2]. The outcome following the onset of acute respiratory distress syndrome (ARDS) remains poor, with an approximately 40% mortality rate [3]. Right ventricular (RV) dysfunction is commonly observed in moderate-to-severe ARDS, and its management remains challenging in view of its complex background, an incomplete understanding of its pathophysiology, and increased mortality. The prevalence of echocardiographically evident RV dysfunction in ARDS ranges from 22% to 50%. Factors adversely affecting RV function include hypoxic pulmonary vasoconstriction [4,5], hypercapnia [6], and invasive ventilation with high driving pressure [7,8,9,10]. During the COVID-19 pandemic, increased rates of RV dysfunction were described in the setting of ARDS, secondary to pulmonary hypertension, including prolonged ARDS and VV ECMO support with the development of microthrombi in the pulmonary vasculature [11]. ECMO as a bridge to lung transplantation (LTx) is an established approach for refractory ARDS and end-stage lung disease in the context of pulmonary hypertension (PH) and RV dysfunction [12,13,14,15,16]. An alternative approach for RV support is the use of ProtekDuo, which is a single-site dual-lumen cannula for percutaneous insertion through the right internal jugular vein with the placement of its tip in the main pulmonary artery [17,18]. The proximal fenestration of the cannula drains venous blood from the right atrium (RA) to the ECMO system and re-infuses it into the main pulmonary artery through distal fenestrations. This configuration, namely (dl)V-P ECMO, bypasses the RV with the set ECMO flow, leading to suitable and effective RV assistance [11]. The use of VP ECMO may mitigate the development or progression of right ventricular dysfunction and lead to a potentially different outcome, considering that VV ECMO is challenged by the onset of RV dysfunction [19,20,21,22] and may require reconfiguration to V-P ECMO [23]. The addition of an oxygenator membrane lung (ML) provides VV ECMO support with reduced potential for recirculation. Limited data suggest that the groin-free trans-jugular insertion is associated with shorter procedural time, faster weaning time, and reduced incidence of complications, such as bleeding and infection, with faster mobilisation time and reduced risk of re-operation. Single-site dual-lumen VP ECMO cannulation has been used to provide haemodynamic support in different settings, such as high-risk cardiac surgery, post-cardiotomy cardiogenic shock, acute decompensated heart failure, and acute respiratory failure [8,24,25]. Despite versatile and important clinical advantages, the use of the V-P ECMO configuration is still relatively new, and its adoption may be slow and infrequent compared to the standard of care VV ECMO. Although there is wide recognition of the use of VV ECMO support in bridging critical patients to lung transplantation [26], we sought to review the use of ProtekDuo^TM^ (CardiacAssist Inc., Pittsburgh, PA, USA) in this challenging setting and how its role is currently evolving.

## 2. Materials and Methods

**Figure 1:**
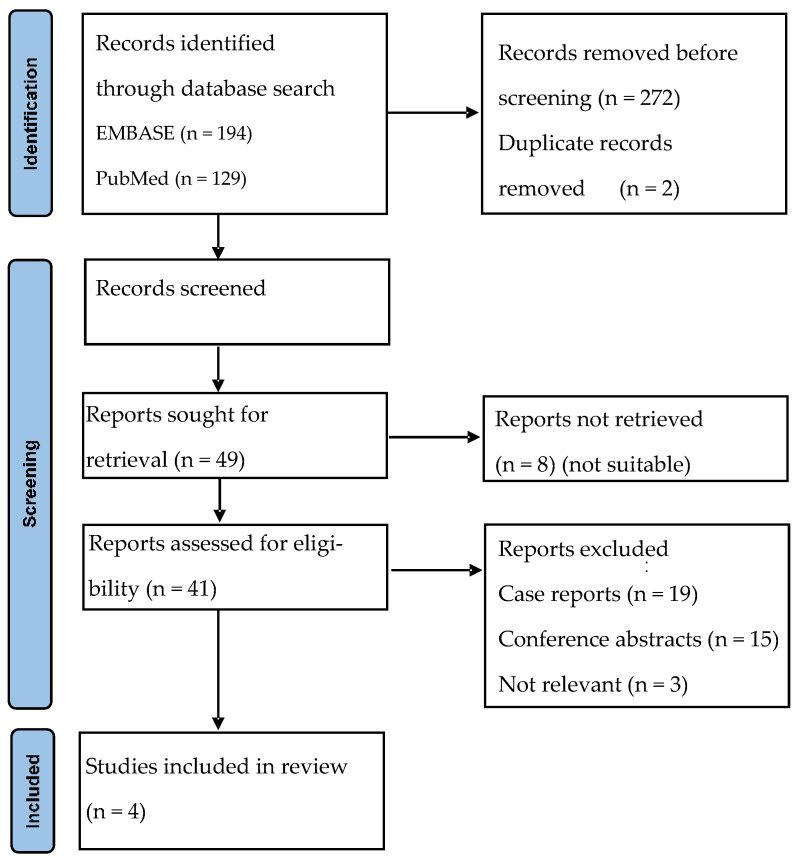
“PRISMA” flow chart.

This systematic review was registered with INPLASY (INPLASY202460053). A web-based literature search on PubMed and EMBASE was conducted. We used the PICOS (Participants, Intervention, Comparison, Outcome and Study Design, Table 1) approach for the selection of clinical studies following our systematic search. The PRISMA (Preferred Reporting Items for Systematic Reviews and Meta-Analyses, Figure 1) approach was considered, the main purpose of which is to help ensure the clarity and transparency of systematic reviews. It was developed using an evidence-based approach, and it is not intended as a quality assessment tool [27]. Subsequently, the PRISMA statement has been further developed to specifically address the reporting of systematic reviews, incorporating network meta-analyses [28]. Finally, PRISMA-P is intended to help the preparation and reporting of a robust protocol for a systematic review [29]. The search strategy included specific terms and their combinations, such as ProtekDuo or percutaneous right ventricular assist device and oxygenator, ECMO or extracorporeal membrane oxygenation, ECLS or extracorporeal life support, and RVAD or RVAD/OXY or oxyRVAD, and transplantation/lung transplantation. Although these terms are not specific and not compliant with guidelines, they are, unfortunately, still in use [30,31]. The literature was screened for any relevant publications on the subject, followed by a redundancy check. All authors contributed to the selection of the eligible articles that would be included in the systematic review. Discordances were addressed by consensus. Clinical guidelines, reviews, book chapters, editorials, and letters to the editor were excluded. Case series, case reports, observational studies, and cohort studies were selected and further reviewed to ascertain their suitability. No randomised prospective trials were available. The Oxford grading system (2009) was used to assess the quality of the selected studies. VP ECMO and patients aged above 18 years old were considered inclusion criteria. VA ECMO and VV ECMO were considered exclusion criteria.

## 3. Results

We identified a total number of 323 publications: 194 in EMBASE and 129 in PubMed. In total, 2 duplicates and 272 publications were removed before screening because they were not relevant to the subject. The remaining 49 publications were screened, following which 19 case reports (less than three patients) and 15 conference abstracts were removed. Additionally, 11 other publications were removed because they were not suitable for the purposes of the review. The final review included four suitable articles, as listed in Table 2.

Four case series reporting single-centre experience with a total of 34 patients were included in this review [32,33,34,35].

One case series included eight patients bridged to lung transplantation with percutaneous VP ECMO [32]. One case series included patients placed on what was called “oxyRVAD” with a (dl)VP ECMO cannula as a bridge to lung transplantation [33]. Two case series included patients on “oxyRVAD” as a bridge to lung transplantation without dual-lumen cannulas [34,35]. The studies had no control groups or measured outcomes in a blinded manner. The articles fulfilled grades C and D of evidence according to the Oxford grading system (2009).

## 4. Discussion

Our analysis of the four available articles revealed the use of VP ECMO in 34 patients with end-stage lung disease and concomitant right ventricular failure requiring support as a bridge to lung transplantation. The duration of support was variable, ranging from a median of 42 h to a median of 15 days. The bridge to lung transplant was achieved for between 71% and 100% of cases. Survival to hospital discharge was 90% to 100%. One-year and two-year survival was 80% and 75%. Technical improvements in ECMO support have allowed the expansion of its role to lung transplantation, with particular reference to the membrane lung (ML) using polymethylpentene (PMP), pump configuration (centrifugal), and circuit developments in association with peripheral percutaneous approaches, including dual-lumen single-site cannulation using the Avalon Elite Bi-Caval Dual Lumen Catheter, Crescent Jugular Dual Lumen Catheter, and more recently, ProtekDuo cannulas. The traditional role of ECMO as a rescue procedure following early graft dysfunction after a lung transplant has evolved to include the whole journey from bridge to transplant to rescue post-transplant [36,37,38]. Lung transplantation has increased in the setting of end-stage lung diseases, such as cystic fibrosis, interstitial lung disease, and chronic obstructive lung disease. Nevertheless, the concerning mortality rate on the waiting list has driven the search for alternative strategies to support this challenging group of patients. The avoidance of mechanical ventilation and the concept of “awake” ECMO as a bridge to transplant allows active involvement in the rehabilitation program with the counteraction of ICU-acquired weakness, ventilator-induced lung injury (VILI), or ventilator-acquired pneumonia [39,40]. Although there are no randomised controlled trials, a previous meta-analysis of retrospective studies reported a 1-year survival rate between 50% and 90%, which was significantly better during spontaneous breathing or an ECMO bridge duration of less than 14 days [41]. ECMO used intraoperatively may be required at any stage when hypoxia, hypercapnia, and haemodynamic instability develop. ECMO can stabilise the haemodynamics and prevent “first lung syndrome” during bilateral lung transplant, namely the hyperperfusion of the first implanted lung during the insertion of the second one. Additionally, it can enhance a protective ventilation strategy and counteract the reperfusion syndrome during one-lung ventilation, lung size mismatch, auto-PEEP, and dynamic hyperinflation [42]. Postoperatively, the main role of ECMO support remains as one of the rescue procedures following early graft failure after lung transplant [26,36,43,44]. The role of ProtekDuo in the setting of lung transplantation with reference to RV failure, secondary to end-stage lung disease, is relatively new and not completely defined. The advancement in percutaneous cannulation techniques has made the V-P ECMO configuration an attractive and perhaps more suitable option in the setting of lung transplantation. It is worth reiterating the fact that the term “OxyRVAD” is not an appropriate one, nor is it defined, and should be replaced by VP or (dl)VP ECMO, depending on the cannula used when referring to this type of approach. We shall continue to use the appropriate term VP ECMO regardless. This configuration stabilises the RV, avoids recirculation with direct delivery of pulmonary artery oxygenation, maintains haemodynamic stability, and offers groin-free mobilisation, rehabilitation, and ventilation weaning. The most recent case series [32] is completely focused on the use of VP ECMO with all patients fully awake and mobile at the time of transplantation. Remarkably, all of them underwent successful lung transplantation, leading to hospital discharge. The case series by Harano et al. [33] describes the use of a (dl)VP ECMO cannula for combined ventricular and respiratory failure with a view to bridging patients to lung transplantation. The series is a retrospective analysis of seven patients with idiopathic pulmonary fibrosis requiring extracorporeal life support (ECLS) as a bridge to lung transplantation. Five patients were successfully bridged to bilateral sequential lung transplant: one patient required VA ECMO; four patients were placed on VP ECMO using ProtekDuo connected to a Tandem Heart or Centrimag pumps. Two VP ECMO patients were extubated and remained ambulant while waiting for a donor offer, with a median ECMO duration of 42 h. The series by Lee et al. [34] includes eight patients with interstitial lung disease who were bridged to lung transplantation on VP ECMO because of right ventricular dysfunction. Seven patients were successfully transplanted after an average VP ECMO duration of 15 days. All of them underwent rehabilitation whilst on VP ECMO. All patients were discharged with a survival rate of 100% at 30 days and 85.7% at 180 days. Lee et al. [35] reported a retrospective review of 14 patients bridged to lung transplant on VP ECMO due to severe right heart dysfunction in end-stage lung disease: eleven with exacerbation of interstitial lung disease; one with COPD; and two with chronic lung allograft dysfunction (CLAD). All patients underwent active rehabilitation on ECMO. Ten patients underwent successful lung transplants with 90% hospital discharge and an 80% one-year survival rate.

Other configurations are available confirming the versatility of ProtekDuo, such as venovenous-pulmonary (VV-P) ECMO with an additional 25 Fr drainage cannula in the femoral vein and veno-venopulmonary (V-VP) ECMO with an additional 25 Fr drainage cannula in the femoral vein for sole drainage and double lumen return through both lumen of the ProtekDuo cannula [19,25,45]. The main feature of this device remains its insertion through the right internal jugular vein using a Seldinger technique under transesophageal echocardiography or fluoroscopy guidance, leading to early patient mobilisation [46]. This allows direct placement in the pulmonary artery in a minimally invasive manner, with effective bypass and decompression of the right ventricle when the pump system is connected. The concomitant insertion of a pulmonary artery catheter for haemodynamic monitoring is possible. Potential risks during ProtekDuo insertion include perforation, damage to the tricuspid and pulmonary valves, pericardial tamponade, and superior vena cava syndrome [47,48]. The transplant setting can be unpredictable, and waiting times can be variable despite being on the urgent or super urgent list when device support is commenced. Therefore, the ability to maintain patients who are awake and mobile while bridging the gap to lung transplantation makes VP ECMO an attractive option. In this context, Ricks et al. described ProtekDuo^®^ when inserted preoperatively for RV protection with VP ECMO in the (dl)V-P ECMO configuration. Intraoperatively, it provided venous drainage for VA ECMO in the (dl)VP-/AO configuration for bilateral orthotopic lung transplantation (BOLT). Postoperatively, the patient remained on (dl)V-P ECMO for RV support and was decannulated with mild RV dysfunction after 5 days. This is the first description of ProtekDuo^®^ used in the (dl)V-P to (dl)VP-/AO to (dl)V-P configuration for the entire perioperative period of BOLT and shows the versatility of this cannula [38].

Despite limited evidence at present, the V-P ECMO configuration with the use of ProtekDuo has the potential to become a game changer in bridging patients with end-stage lung disease and the onset of right ventricular dysfunction to lung transplantation. Increasing awareness of its potential may help spread its use and obtain additional evidence of its effectiveness. Liaison with ELSO would be the way forward to develop future trials, resulting in appropriate guidelines.

## 5. Conclusions

Despite the present limited evidence, the use of ProtekDuo has become very promising for the management of end-stage lung disease as a bridge to lung transplantation. Perseverance may increase its awareness and usage.

## Figures and Tables

**Table 1:** “PICOS” approach for the selection of clinical studies following the systematic search.

**Participants**	Patients undergoing ECMO support as a bridge to lung transplant using ProtekDuo device
**Intervention**	Dual lumen veno-pulmonary ECMO support
**Comparison**	Comparison with those patients who did not require ECMO support
**Outcome**	If ECMO support with ProtekDuo made a difference
**Study Design**	Inclusion of prospective and retrospective clinical studies, and case series. Exclusion of case reports/series with less than 3 patients, editorials, review articles, letters, abstracts, and any non-peer-reviewed publications

**Table 2:** Relevant articles with key information and outcomes: ECMO = extracorporeal membrane oxygenation; VP ECMO = veno-pulmonary ECMO; LTx = lung transplant; OxyRVAD = oxygenator with right ventricular assist device; ECLS = extracorporeal life support; BTT = bridge to transplant; BLTx = bilateral sequential lung transplant; VA ECMO = veno-arterial ECMO; VV ECMO = veno-venous ECMO; VAV ECMO = veno-arterial-veno ECMO; RHF = right heart failure; PA = Pulmonary Artery; ARF = acute respiratory failure; DL RIJV cannula = dual Lumen right internal jugular vein cannula.

Author, Year	Indication	Configuration	ECMO Patients	Support Duration	Outcome
Usman, 2024 [32]	Bridge to lung transplantation	Percutaneous VP ECMO	8 pts Awake and mobilised		All pts LTx All survived to hospital discharge
Harano, 2022 [33]	Bridge to lung transplantation	Dual-lumen PA cannula (ProtekDuo)	7 pts with idiopathic pulmonary fibrosis on ECLS; 2 failed BTT; 5 successful BTT; 1 pt on VA ECMO; 4 pts on VP ECMO; 2 pts extubated and ambulated while waiting for LTX; 2 pts converted to VAV ECMO	Median waiting time on ECLS: 42 h	5 pts BLTx
Lee, 2022 [34]	Bridge from RHF to lung transplant	No dual-lumen PA cannula used	8 pts with interstitial lung disease	Average ECMO duration: 27 days Average duration: 15 days	7 pts underwent LTx.
Lee, 2021 [35]	Bridge to lung transplant in severe RHF due to end-stage lung disease	OxyRVAD	14 pts on OxyRVAD	VV ECMO duration: 21.5 days (median) OxyRVAD duration: 8 days (median)	10 pts bridged to LTx

## Data Availability

Additional data are not available.
